# Typhoid Vaccine in the Treatment of Typhoid Fever

**Published:** 1912-07

**Authors:** Marvin B. Grace

**Affiliations:** Seguin, Texas


For Texas Medical Journal.

### Typhoid Vaccine in the Treatment of Typhoid Fever.—Report of Cases.*

BY MARVIN B. GRACE, M. D., SEGUIN, TEXAS.

Requesting the indulgence of those present for a few moments, I wish to report the following cases with results of treatment by

*Read before the Fifth District Medical Society, San Antonio, Texas, April 4, 1912.

the typhoid vaccine. Prefacing these remarks by the statement that no one realized more than the writer that six swallows do not make a spring. However, they do indicate that the cold, gloomy winter days are numbered, and that the sun will soon shed her radiant smile and dispel the gloom around us.

It has been demonstrated beyond question, both in army work and private practice, that a culture of typhoid bacilli in buillon, after attaining a certain strength as to numbers of bacilli present when subjected to a temperature of 60 degrees C. for one hour for the purpose of killing the bacilli, is an efficient prophylactic treatment for typhoid.

In passing, I beg to report ninety-six persons inoculated with this vaccine during the summer and fall of 1911 at Seguin. Of this number, many were exposed to the disease directly, such as nursing sick members of the same family, and all were exposed to the general cause of the epidemic. Of the number treated, but one, a child aet. 10, which proved an interesting case and the first I treated with vaccine, developed typhoid immediately after first inoculation.

Case No. 1.—D—— B., aet. 10, female. Brother and three sisters in bed with typhoid. The five children and mother convalescing from measles. Had been free of fever six or seven days when typhoid developed in four of the children simultaneously. D. B., her mother and father, the only remaining members of the family out of bed. On June 21st, administered typhoid vaccine to the three remaining up. Either with, or immediately following the inoculation, a temperature of 99$\frac{3}{5}$ occurred, which I ascribed to the reaction following the vaccine. As this temperature recurred on the second and third and soon became continuous, I felt justified in sending her to bed with diagnosis of typhoid.

The nurse's record, which is before me at this writing, shows that she received her second and last injection on July 1st, or ten days following the first. This was followed by a temperature gradually rising to 103 degrees and which declined after six hours. This was the only time her temperature rose above 101 degrees during the entire course of the disease. Temperature remained normal after the sixteenth day. At no time was she seriously ill, or very uncomfortable. Her sisters, one of whom died from general toxemia of typhoid, and brother, none of whom had the vaccine treatment, all ran high temperatures, reaching 105 and 106 degrees at times. All were very sick, one had intestinal hemorrhages, and the duration of all was four to six weeks.

Case No. 2.—Miss E. R., aet. 30, robust; weight 160 pounds. At my first call found her temperature 104 degrees, severe headache, and doing her housework. Tentative diagnosis of typhoid, as this was the fourth day of the disease, so far as the history could show. She was sent to bed, and on the seventh day ran a temperature above 105 degrees, in spite of ice caps and sponging. Typhoid vaccine was used in full dose of 500 million bacteria as first dose. Record shows a decline in temperature after three days had elapsed from the time of the first injection, and six days after temperature was running 99 degrees a. m. to 102 degrees p. m. It looked as if the disease was controlled, but on the tenth day following inoculation an exacerbation occurred. Then begun severe symptoms, rapid pulse, dry glazed tongue, high temperature, abdominal distension, intestinal hemorrhage, mental symptoms, etc. These occurred between the tenth and thirteenth day after the only inoculation up to this time. I must confess I was hesitating on the vaccine but administered it again thirteen days after the first dose, giving the full dose of a 1000 million bacteria. Temperature now came down gradually, other symptoms cleared up, and patient was convalescent on the twenty-fourth day of disease. The medication used in this as in other cases here reported was calomel gr. 1-20, salol gr. 2 and quinine gr. 1, every four hours, with castor oil as needed. I found it necessary to administer morphine and atropine hypodermically at first for headaches.

Case No. 3.—L. B., aet. 19, male. Was seen about third day of disease and found temperature running rather high, and on next day reached 105 degrees, and was extremely hard to reduce. Administered vaccine the evening of the fourth day. On the seventh day of the disease the temperature dropped to 100 degrees a. m. and 102 degrees p. m., where it ran with but little variation until after the next inoculation, which was given seven days following the first. This was followed by a further reduction and an established convalescence on the fifteenth day of the disease.

Case No. 4.—H. A. E., aet. 30, male. Usual prodromal symptoms. Consulting me at office for several days before the nature of the case would justify me in a diagnosis of typhoid. Temperature range was low. Was ordered to bed and vaccine administered on sixth day of the disease. Temperature, which never ran above 101 degrees, subsided soon after the second injection, which was given on the thirteenth day of the disease. Duration about eighteen days.

Case No. 5.—Miss A. B. P., aet. 16. Subject plethoric. Early temperature range was high. Headache and nervous symptoms intense. Administered typhoid vaccine on fourth day of disease. Amelioration of the nervous symptoms came with the early decline in temperature, which begun on the third day following the inoculation and by the fifth day had reduced from 105 degrees p. m. to 101 p. m. Second injection was given seven days after the first and on the eleventh day of the disease. Last temperature on the nurse's chart shows 99 2-10 degrees on the fifteenth day of disease.

Case No. 6.—K. F., aet. 5, female, negro (from memory). Saw patient about one week after beginning of illness. Temperature at time of visit above 103 degrees. Administered typhoid vaccine. About one week later was free of fever. Lost sight of the case until called again ten days later and found a relapse. Again gave vaccine, which was followed by a reduction in temperature. This reinfection lasted fifteen days, during which time two inoculations were made.

When we take into consideration the fact that these cases occurred during an epidemic of a severe type of typhoid, where the mortality was rather high; where the usual duration was from four to six weeks; where but few mild cases occurred, and that I have reported the last six consecutive cases in my practice, that I did not use this treatment on selected cases, it would seem to offer some evidence that some specific action of the vaccine was present. Cases Nos. 2, 3 and 5 all presented the early symptoms of a severe attack. Case No. 1 was rendered mild, in my opinion, by the initial dose at the beginning of the disease. Case No. 4 would probably have been a mild attack under any of the regular methods of treatment.

While I am not yet able to submit my conclusions, reserving these for the results in many consecutive cases, yet I beg to call your attention to the following indications:

1. That typhoid vaccine should be used in the usual prophylactic dose as early as possible in typhoid fever. The first dose for an adult being 500 million, this to be followed in seven days by a dose of one billion of the bacteria. This to again be followed in seven days by a similar dose should the case require it by showing an active process still exists.

2. It is apparent that it does not produce any very unpleasant effects if we except a slight rise of temperature that usually passes off in six or eight hours. Except in rare instances but slight irritation is seen at the sight of injection.

3. Vaccine should be administered subcutaneously in the inter or sub-scapular area, and not in the deltoid region if we wish to avoid considerable pain and axillary tenderness.

4. The treatment does in no way interfere with any special line of treatment, the physician prefers, and seems to offer much when used early in large dosage, for the amelioration and abbreviation of the disease.

5. That it does not take eight or ten days, as seems the case in producing immunity, for it to produce the resisting substances in the blood in a case of typhoid, for we can well imagine this production well under way, stimulated by the living organisms in the body, and that we are merely assisting nature in the task of building up these fighting elements of the blood.

The production of these anti-bodies is probably very rapid in these active cases, especially when the remedy is administered early, hence we may look for results within five days.

It is not within the province of this paper to discuss Erlich's theory or Wright's opsonic index as applied to this important subject. The specific toxins of the typhoid bacillus have, as yet, not been isolated. Until this can be done we will probably have no antitoxin, hence we must go to the resisting powers of the body and ascertain its method of opposing the organism in order to gain a basis for treatment.

Wright has demonstrated that it builds up in the blood, in the process of immunizing man against typhoid, certain substances called agglutinating antitropins, bactericidal or bacteriolytic antitropins, antitoxic antitropins and opsonic antitropins. He emphasizes the first two of these as the principal agents of defense.

The therapeutic application of active immunization is based on the hope of stimulating the body to produce antagonistic substances more rapidly than occurs under the stimulus of the natural infection.

---

An amebic colitis that has been quiescent frequently lights up after a complicating liver abscess has been drained. Such patients may recover from the abscess and succumb to the colitis. In all cases of amebic liver abscess, therefore, treat the bowel, also, by appendicostomy and irrigation, even though it is giving no symptoms.—*American Journal of Surgery.*

